# Successful chemotherapy with continuous immunotherapy for primary pulmonary endovascular epithelioid hemangioendothelioma: A case report

**DOI:** 10.1097/MD.0000000000032914

**Published:** 2023-02-17

**Authors:** Wenliang Guo, Daibing Zhou, Houquan Huang, Haiming Chen, Xiaofeng Wu, Xin Yang, Huiling Ye, Cheng Hong

**Affiliations:** a State Key Laboratory of Respiratory Disease & National Clinical Research Center for Respiratory Disease, Guangzhou Institute of Respiratory Health, The First Affiliated Hospital of Guangzhou Medical University, Guangzhou, China; b Department of Respiratory and Critical Care Medicine, Huashan Hospital, Fudan University, Shanghai, China.

**Keywords:** diagnosis, endovascular neoplasm, epithelioid hemangioendothelioma, MSH2, treatment

## Abstract

Epithelioid hemangioendothelioma (EHE) is a rare, low to moderate-grade malignancy, even less in pulmonary endovascular neoplasm. Patients with pulmonary EHE have no optimal treatment, resulting in poor prognoses.

**Patient concerns:**

We reported a 42-year-old man with multiple mild metabolic uptakes in pulmonary endovascular filling defect with a maximum standardized uptake value of 4.5 by 18-fluorodeoxyglucose/fibroblast associated protein inhibitor-positron emission tomography/ computed tomography. Anticoagulant treatment was not effective with the diagnosis of acute pulmonary embolism.

**Diagnoses:**

A primary endovascular EHE pulmonary endovascular epithelioid hemangioendothelioma was diagnosed by endovascular biopsy with positive stains for molecular CD31, CD34 and CAMTA1, and it had low proliferative capacity characterized by Ki-67 of 5%. The mutation gene MSH2 (p.Y656 in exon 12) (mutation abundance of 0.07%) from peripheral blood indicates the potential benefit of an immune checkpoint inhibitor, pembrolizumab.

**Interventions and outcomes:**

The patient was treated with tri-weekly paclitaxel (175mg/m2) and carboplatin (AUC 5) chemotherapy regimen. He exerted a remarkable response after 5 cycles (21 days per cycle) and Pembrolizumab (200mg once monthly) as maintenance treatment.

**Lessons:**

This case highlights the diagnostic challenge of differentiating endovascular lesions and optimal therapy for pulmonary EHE. Importantly, it indicated that the mutation gene MSH2 (p.Y656) might influence the pathogenesis of EHE.

## 1. Introduction

Pulmonary epithelioid hemangioendothelioma (P-EHE) is a rare vascular tumor. The heterogeneous nature of epithelioid hemangioendothelioma (EHE) varies between these indolent diseases and aggressive malignancies. It could be a misdiagnosis of endovascular sites, like chronic pulmonary embolism and pulmonary artery sarcoma were hard to discern with EHE.^[1]^ P-EHE could present numerous features, including lymph node involvement, perivascular nodules, pleural effusion, and reticulonodular and wedge-shaped infarcts.^[2]^ The primary site, involved organs and tumor sizes are related to the prognosis. Endovascular biopsy carries an extremely high risk and results in delayed diagnosis and optimal treatment.

Although treatment for EHE has chemo-/immune-therapy and antiangiogenic drugs, it is still confined to case series without available large randomized trials. Bevacizumab-based therapy was reported in metastatic hepatic EHE.^[3]^ Reports of optimal combination regimens and consensus on standard treatment strategies for pulmonary endovascular EHE pulmonary endovascular epithelioid hemangioendothelioma (PE-EHE) are still needed. Herein, we reported a rare case of PE-EHE treated with chemotherapy and recompense pembrolizumab as maintenance treatment.

## 2. Case report

A 42-year-old man was presented to the local hospital with a history of unexplained chronic dyspnea accompanied by intermittent cough, sputum and endurance decline. He was a nonsmoker and nonalcoholic with no other potable medical history. Anticoagulant treatment was not effective with the diagnosis of acute pulmonary embolism. Thus, he was admitted to our hospital on January 6, 2022.

**Figure 1.**
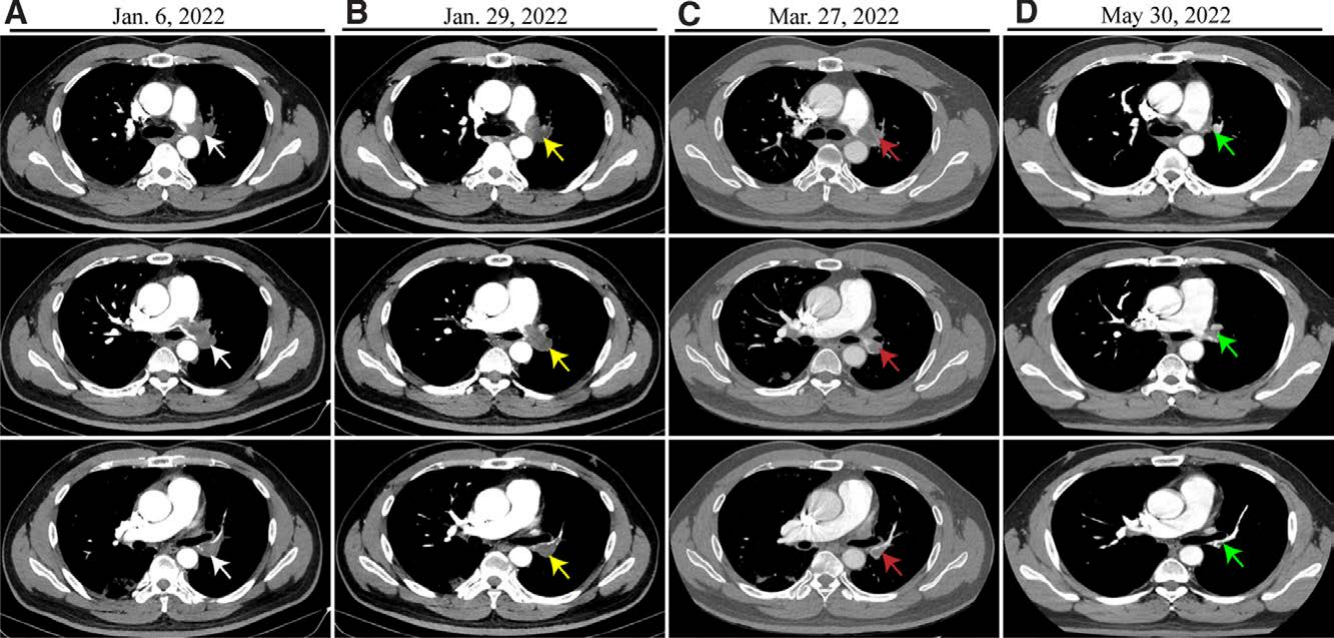
Thoracic CTPA scan images before and after therapy. (a) CTPA scan on admission (white arrow). CTPA images indicated that the endovascular lesions reduced gradually before the second (b) (yellow arrow), the third (c) (red yellow) chemotherapy and after the fifth (d) (green arrow) chemotherapy. Three images represent 3 different frames from the upper to the down. CTPA = Computed tomography pulmonary angiography.

**Figure 2.**
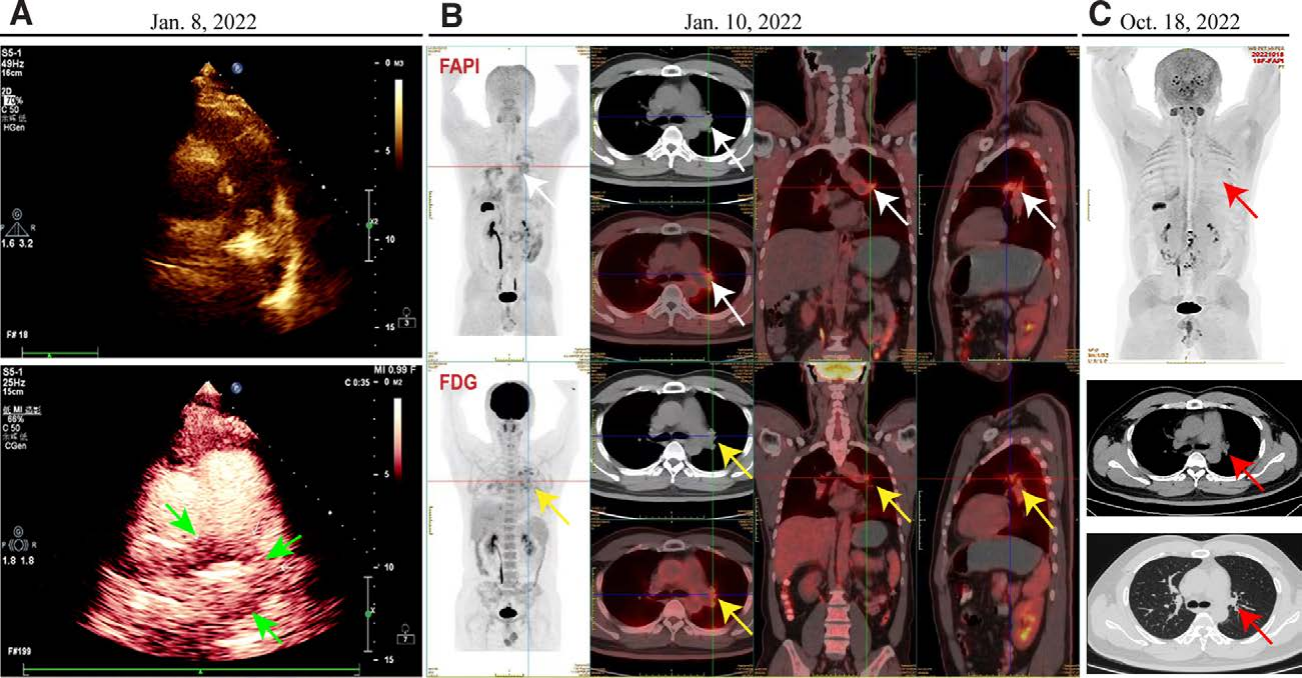
Visualization of the pulmonary endovascular lesions with ^18^F-FDG/FAPI-PET/CT and right heart contrast echocardiography. (a) right heart contrast echocardiography showed a solid mass with abundant blood provision in the pulmonary artery (green arrow). (b) Before diagnosing PE-EHE, ^18^F-FDG/FAPI-PET/CT presented an endovascular lesion with a SUVmax of 4.5 in the left main vessels in the 3-dimensional fluoroscopy, transverse, sagittal plane and coronal planes (from left to right, respectively). the white arrow represent the 18F-FAPI-PET/CT, and the yellow arrow for the 18F-FDG -PET/CT. (c) ^18^F-FAPI-PET/CT revealed the SUVmax of the left pulmonary vascular lesion reduced to the background level (red arrow). ^18^F-FDG/FAPI-PET/CT = 18-fluorodeoxyglucose/fibroblast associated protein inhibitor-positron emission tomography/ computed tomography, PE-EHE = pulmonary endovascular epithelioid hemangioendothelioma, SUVmax = maximum standardized uptake value.

**Figure 3.**
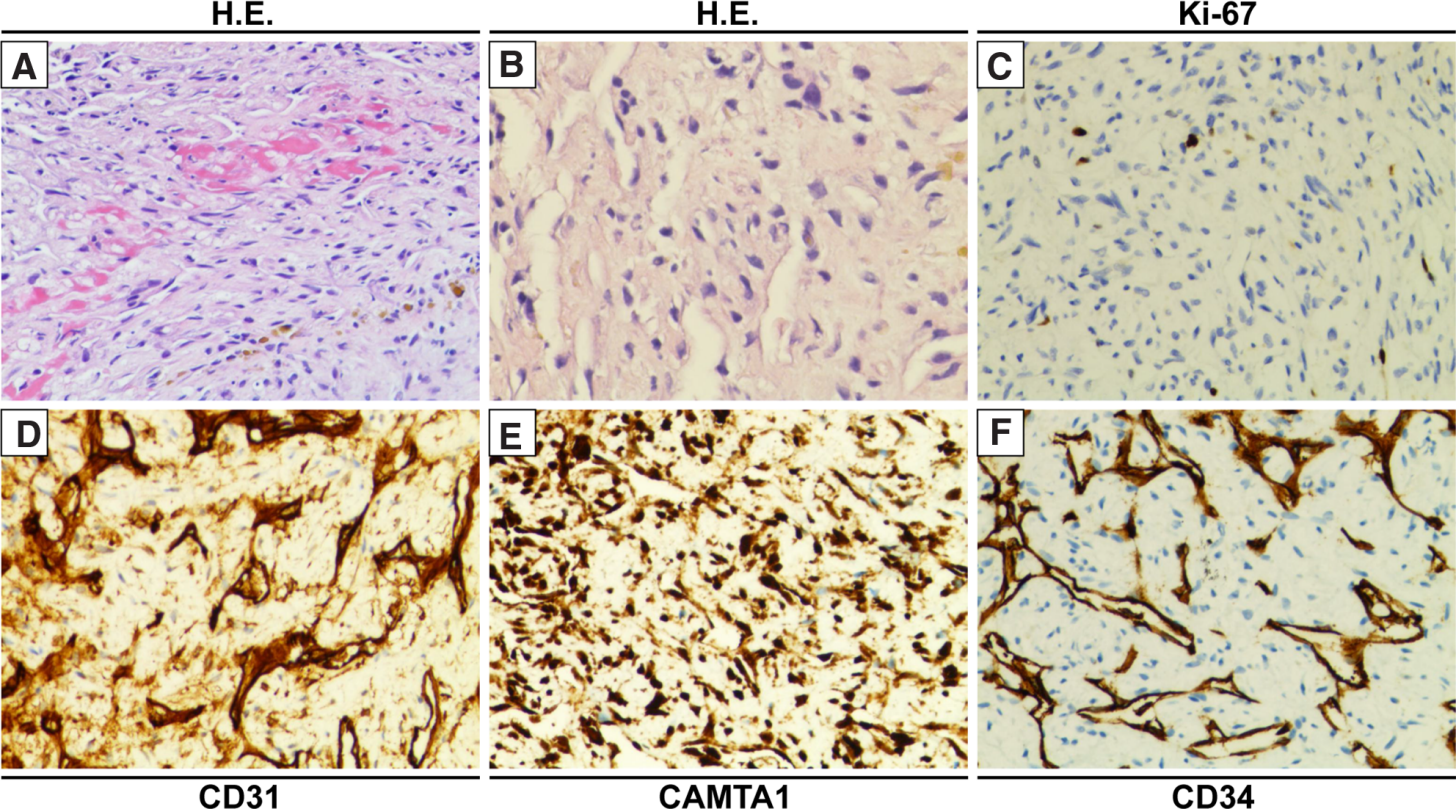
Histopathological characteristics of the PE-EHE. (a) and (b) the specimen from the endovascular site presented vessels of different sizes, irregular anastomosis, and spindle-shake tumor cells; the cytoplasm of tumor cells was clear. Immunostaining showed a low proliferative activity of Ki67(c) and tumor cells positive for CD31(d), CAMTA1(e), and CD31(f). (a), (c), (d), (e) and (f) magnification, x200; (b) magnification, x400. PE-EHE = pulmonary endovascular epithelioid hemangioendothelioma.

The results of blood tests were as follows: White blood cell 6.6 × 10^9 (normal range, 4-10 × 10^9 cells/L), C-reaction protein 3.1(normal range, 0–0.6mg/dL); lactate dehydrogenase (LDH) 255.2 (normal range: 109–255 U/L); D-dimer 1862 (normal range: 68–494 ng/mL EU), Homocysteine 11.93 (normal range: 5–15 μmol/L); NSE 17.92 (normal range: 0–16.3 ng/mL), CA125 115.5 (normal range: 0–16.3 U/mL), CEA 2.51 (normal range: 0 - 5 ng/mL), CYFRA21-1 1.95 (normal range: 0–3.3 ng/mL). Computed tomography pulmonary angiography (CTPA) scan revealed incomplete occlusion of left pulmonary arteries without enlargement of hilar lymphatic nodules (Fig. 1a). However, bronchoscopy and bronchoalveolar lavage fluid had no sign of malignancy and inflammation. Right heart contrast echocardiography showed a solid mass in the pulmonary artery with abundant blood provision (Fig. 2a). 18-Fluorodeoxyglucose/fibroblast associated protein inhibitor-positron emission tomography/ computed tomography indicated intraluminal lesions with invasive growth near the vessel, and the maximum standardized uptake value (SUVmax) was 4.5(Fig. 2b). It suggested a pulmonary intraluminal tumor with thrombosis. Given the potential risk of malignancy based on the patient age, the Ultrasonic cardiogram and the 18-fluorodeoxyglucose/fibroblast associated protein inhibitor-positron emission tomography/ computed tomography characteristics of the lesion, a percutaneous transluminal pulmonary biopsy was performed. A hematoxylin and eosin (H.E.) stain of the lesion microscopically revealed vessels of different sizes, irregular anastomosis, spindle-shake tumor cells, and the cytoplasm of tumor cells was transparent and covered with red blood cells (Fig. 3a–b). The cells showed low proliferation activity, with only 5% immune-positive cells for the proliferation marker Ki67(Fig. 3c). Additional immunostaining showed positive staining for biomarkers CD31, CAMTA1 and CD34 (Fig. 3d–f). These histopathological findings demonstrated a diagnosis of primary PE-EHE.

The patient performed a response after tri-weekly paclitaxel (175mg/m2) and carboplatin (AUC 5) chemotherapy regimen for 5 cycles (21 days per cycle) (Fig. 1b–d). In addition, we collected peripheral blood samples for whole-genome sequencing. It had a positive result for mutation gene MSH2 (p.Y656 in exon 12), negative for immune checkpoint marker PD-1/PD-L1 but also presented potential benefits for the patient based on the treatment of pembrolizumab. Thus, the patient was treated with pembrolizumab (200mg once monthly) as a maintenance treatment. The ^18^F-FAPI-PET/CT showed that the SUVmax of FAPI in the left pulmonary vascular wall reduced to the background level and was considered inhibited (Fig. 2c). The patient is a follow-up. Informed consent for all data and clinical history was obtained from the patient.

## 3. Discussion

It is a rare scenario of PE-EHE in a male patient who initially presented with acute pulmonary embolism, then performed anticoagulant therapy, and was again admitted to our hospital as a progressive sign of vascular aggressivity near endovascular lesions. EHE is a rare vascular tumor with indolent clinical behavior, sometimes performed with low to moderate aggressiveness. It commonly occurs in patients aged 30 to 50 years. Pulmonary EHE performed a female predominance, with a median age of 40.^[4,5]^ It reported that pleural EHE was more likely to occur in males aged 31 to 80.^[6]^ Patients with weight loss, anemia, pleural hemorrhagic effusions and pulmonary symptoms had a poor prognosis.^[5]^

The pathogenesis of EHE remains unclear. Angiogenic stimulators promoted endothelial cell proliferation and resulted in uncontrolled growth.^[7]^ A recurrent t (1;3) (p36.3; q25) chromosomal translocation, YAP1-TFE3 fusion, and WWTR1-CAMTA1 rearrangements in EHE patients drive the tumor biology, and a recent study showed that interaction of the ATAC complex with YAP1-TFE3 and WWTR1-CAMTA1 fusion plays a pivot role in the oncogenicity.^[8,9]^ It indicates a unifying enzymatic therapy target for EHE. Additionally, Bartonella infection also induced the high expression of vascular endothelial growth factor (VEGF) and stimulated the uncontrolled proliferation of vessels.^[10]^ It revealed that a high-risk EHE characterized by VEGF expression and its receptors provide the potential for anti-VEGF therapy. New biomarkers of TP53, murine double minute-2 and decreased caveolin-1 would be verified in the development of EHE.^[11]^ Recently multi-mutation in functions like DNA repair, epigenetic modification and cell cycle dysregulation influence the pathogenesis,^[12]^ just as the mutation of MSH2 in this case, which represents a regulator of mismatch DNA repair. It revealed that MSH2 might influence the pathogenesis of PE-EHE. However, whether the status of MSH2 mutation affects the efficacy of pembrolizumab against PE-EHE remains unclear.

It presented difficulty in distinguishing it from pulmonary and endovascular diseases. CTPA and 18FDG-PET/CT scan strategies could improve diagnostic accuracy. In our previous studies, CTPA had a positive predictive value (PPV) of 100% in pulmonary endovascular masses if it presented at least 5 signs. Given a CT scan exhibiting no more than 4 abnormal signs, ^18^FDG-PET/CT could improve the accuracy of uncertain pulmonary masses if the pulmonary masses had a value of SUVmax > 3.4.^[13]^ In this report, the ^18^FDG-PET/CT indicated an uptake value of SUVmax 4.5 in intraluminal lesions with invasive growth near the vessel, in which the SUV values were close to the average levels of benign disease. Furthermore, the low uptake of SUVmax by ^18^FDG-PET/CT in endovascular lesions is consistent with immunohistochemistry low proliferative activity of Ki-67. A review revealed that the average SUVmax for pulmonary EHE was 3.0 (range 1.2–8.5).^[14]^ It indicated that pulmonary endovascular EHE had a low proliferative capacity. Given that endovascular biopsy presents an extremely high risk, like massive hemorrhage, vascular perforation and distal embolization. It presents an extensive diagnostic challenge.

Therapeutic options for pulmonary EHE are limited, including resection of primary and metastatic tumors, radiotherapy, organ transplantation, chemotherapy, and immunotherapy, and radiotherapy may be considered a neoadjuvant therapy for unresectable disease.^[15]^ The study found that the 5 over-survival patients with multifocal hepatic EHE after liver transplantation exceeded 50%.^[16]^ As for unresectable or metastatic EHE, recommended strategies are challenging. The median overall survival in those with inoperable hepatic EHE exceeded those with lung or mediastinal disease.^[17]^ According to the guidance of soft tissue sarcomas, cytotoxic chemotherapy for EHE, as a primary or complementary strategy, included anthracyclines, paclitaxel, 5-fluorouracil, pemetrexed, cyclophosphamide, etoposide, ifosfamide and platinum-based regimens.^[16,17]^ Cytotoxic therapies aim to be available with disease stabilization and surgical resection. Compared with the primary site outside the liver and bone, the occurrence of pulmonary EHE is rare. Pulmonary EHE presents with multiple or bilateral lesions. Surgery can be performed for unilateral nodules, and lung transplantation could be evaluated if patients present with pulmonary vascular aggressivity, hemorrhagic pleural effusion and anemia.^[5]^ Of some asymptomatic patients without significant organ involvement, they performed partial spontaneous regression.^[18]^ In this case, we demonstrate a patient with PE-EHE and the mismatch repair gene MSH2 (p. Y655) mutation of low abundance of 0.07%, which could be oncogenicity and response to PD-1 therapy.^[19]^ Of course, we recommended the tri-weekly strategies with carboplatin and paclitaxel chemotherapy regimen; the patient exerted a remarkable response after 5 cycles (21 days per cycle). According to the results of ctDNA in serum, Pembrolizumab might be effective. Thus, we disable the chemotherapy regimens and recompense pembrolizumab as a maintenance treatment. The patients performed well in the perspective of the present scenario. This case highlights a rare case with PE-EHE and a worth considering strategies of combination treatment with chemo-and immune therapy for those comparable patients.

In summary, PE-EHE is a rare, malignant vascular tumors. The radiology of endovascular EHE could be highly variable because of being misdiagnosed with pulmonary artery sarcoma and tumor thrombus with acute pulmonary embolism. ^18^FDG-PET/CT scan could improve diagnostic accuracy. This case provides a new treatment strategy for pulmonary endovascular EHE.

## Author contributions

**Conceptualization:** Wenliang Guo, Daibing Zhou, Houquan Huang, Cheng Hong.

**Data curation:** Haiming Chen, Xiaofeng Wu, Xin Yang, Huiling Ye.

**Formal analysis:** Haiming Chen, Xiaofeng Wu, Xin Yang, Huiling Ye.

**Funding acquisition:** Cheng Hong.

**Investigation:** Haiming Chen, Xiaofeng Wu, Xin Yang, Huiling Ye.

**Resources:** Cheng Hong.

**Supervision:** Cheng Hong.

**Visualization:** Wenliang Guo, Daibing Zhou, Cheng Hong.

**Writing - original draft:** Wenliang Guo, Daibing Zhou, Houquan Huang.

**Writing - review & editing:** Cheng Hong.
